# Tumor stroma as contributing factor in the lymph node metastases process?

**DOI:** 10.18632/oncotarget.26644

**Published:** 2019-01-29

**Authors:** Wilma E. Mesker, Gabi W. van Pelt, Rob A.E.M. Tollenaar

**Affiliations:** Wilma E. Mesker: Department of Surgery, Leiden University Medical Centre, Leiden, The Netherlands

**Keywords:** tumor-stroma, lymph nodes, metastases, histology, heterogeneity

Tumor associated stroma as part of the tumor micro-environment has increasingly gained interest and acceptance in the field of patients prognostication and treatment. Stroma is not the innocent bystander as previously thought but co-orchestrates the metastases process. The amount of stroma in the primary tumor (PT) is a strong prognostic parameter for breast, colon and other epithelial malignancies. The so called tumor-stroma ratio (TSR) distinguishes between patients with good and worse outcome of disease [1-4].

The presence of tumor cells in lymph nodes (LN) is important for clinical decision making. In recent papers we have shown that tumor associated fibroblasts are present in high amounts in the lymph nodes from patients with colon and breast cancer [3, 5]. The high stromal scores (>50% per image field) correspond with the aggressive behavior of the tumor. Patients with a high amount of stromal cells in one or more lymph nodes showed a worse overall and disease free survival. Patients with a low amount of stroma showed statistically significant good outcomes. What was surprising is that the observed metastases process was heterogenous, that will say: some lymph nodes were occupied with less tumor cells but many fibroblasts, whereas also the opposite was observed within the same patient. Based on these findings we might say that tumor associated fibroblasts have the capacity to metastasize or can accompany metastasizing tumor cells. However, as in some cases only fibroblasts were seen in mostly tumor-free lymph nodes, speaks against the last option.

This strong heterogeneity within the metastasizing process of the stroma was observed using microscopical investigation of routine stained tissue slides but other studies have investigated expression levels on the molecular level and validated our findings [6-9]. In studies to determine prognostic markers for colorectal cancer and investigating the corresponding LN metastases, the expression patterns of some of the markers showed to be heterogeneous between the PT and LN metastases. While the expression of p53 has been documented to be similar between PT and LN metastases, the EGFR and HER2 expression differed significantly [6, 8, 9]. This differences in EGFR and HER2 expression indicate that the PT does not accurately reflect the metastasis situation and we need the information of the LN metastases, which might have important clinical implications.

For colon cancer stage III it was shown that the analysis of the TSR in metastatic LNs is of additional value with respect to survival time of the patients and can be considered as guide for selective treatment to overcome over- and undertreatment [3].

Breast cancer patients with LN metastases were previously immediately eligible for adjuvant chemotherapy, irrespective of other clinic-pathological parameters. As studies have shown that patients with 1-3 positive LNs do not necessarily have a worse prognosis compared to node-negative tumors, subsequent guidelines have since stated that LN involvement in itself is not a reason for adjuvant chemotherapy. Analogous to our work regarding the prognostic implication of stromal proliferation in PTs, we investigated the added significance of assessing stroma in breast cancer positive LNs. We found that incorporating the TSR of LNs combined with the TSR of the corresponding PT provided a superior prediction of relapse free period (RFP) and a group of patients with a notably high risk could be identified. The fact that this patient group showed a recurrence rate of 92% after 10 years, considers this method most capable of identifying patients with a worse prognosis [3].

An interesting observation is the strong discrepancy between TSR in the PT with those of the LNs of the same patient. In more than 50% of the patients heterogeneity was observed between the stroma percentage category in the PT and LNs. This finding might be reflective of differential activity of the signaling processes across primary and metastatic tumors. The formation of genetically and transcriptionally distinct sub clones of tumor cells that arise during tumor evolution might have an influence on both the activation of tumor-associated stroma as well as tumor cell dissemination.

Taking tumor heterogeneity into consideration the TSR might be used as a marker to specifically select patients for therapy. Mechanisms of therapeutic resistance were recently identified, which were mainly conferred by changes in the tumor microenvironment. For future patient treatment regimens this might indicate the development of new therapies targeting the non-cancer stromal cells [10].

Incorporating the TSR in clinical practice has clear advantages compared to other potential biomarkers. TSR scoring can be carried out on standard H&E slides and is performed by visually eyeballing of the tissue during standard pathological assessment. TSR scoring takes less than a minute and requires no additional costs. Implementation of this method in daily practice is therefore an easy and non-expensive option.
